# A novel visual brain-computer interfaces paradigm based on evoked related potentials evoked by weak and small number of stimuli

**DOI:** 10.3389/fnins.2023.1178283

**Published:** 2023-06-05

**Authors:** Xiaolin Xiao, Runyuan Gao, Xiaoyu Zhou, Weibo Yi, Fangzhou Xu, Kun Wang, Minpeng Xu, Dong Ming

**Affiliations:** ^1^School of Precision Instruments and Optoelectronics Engineering, Tianjin University, Tianjin, China; ^2^Academy of Medical Engineering and Translational Medicine, Tianjin University, Tianjin, China; ^3^Beijing Institute of Mechanical Equipment, Beijing, China; ^4^International School for Optoelectronic Engineering, Qilu University of Technology, Shandong Academy of Sciences, Jinan, China

**Keywords:** v-BCI, weak stimuli, evoked related potential, discriminative spatial patterns, information transfer rate

## Abstract

**Introduction:**

Traditional visual Brain-Computer Interfaces (v-BCIs) usually use large-size stimuli to attract more attention from users and then elicit more distinct and robust EEG responses, which would cause visual fatigue and limit the length of use of the system. On the contrary, small-size stimuli always need multiple and repeated stimulus to code more instructions and increase separability among each code. These common v-BCIs paradigms can cause problems such as redundant coding, long calibration time, and visual fatigue.

**Methods:**

To address these problems, this study presented a novel v-BCI paradigm using weak and small number of stimuli, and realized a nine-instruction v-BCI system that controlled by only three tiny stimuli. Each of these stimuli were located between instructions, occupied area with eccentricities subtended 0.4°, and flashed in the row-column paradigm. The weak stimuli around each instruction would evoke specific evoked related potentials (ERPs), and a template-matching method based on discriminative spatial pattern (DSP) was employed to recognize these ERPs containing the intention of users. Nine subjects participated in the offline and online experiments using this novel paradigm.

**Results:**

The average accuracy of the offline experiment was 93.46% and the online average information transfer rate (ITR) was 120.95 bits/min. Notably, the highest online ITR achieved 177.5 bits/min.

**Discussion:**

These results demonstrate the feasibility of using a weak and small number of stimuli to implement a friendly v-BCI. Furthermore, the proposed novel paradigm achieved higher ITR than traditional ones using ERPs as the controlled signal, which showed its superior performance and may have great potential of being widely used in various fields.

## 1. Introduction

Brain-Computer Interfaces (BCIs), which provide a new communication pathway between humans and the outside world, and received great attention in recent years (Lebedev and Nicolelis, 2006; Wolpaw, 2014). Among the various BCIs, the electroencephalogram (EEG) based BCI has become a popular solution in BCI research, which is considered non-invasiveness, low-cost, and more convenient to set up (Xu et al., 2013; Chen et al., 2015). Notably, visual BCIs (v-BCIs) can achieve higher information transfer rate (ITR) than other EEG-BCIs, due to the high signal-noise rate (SNR) of visual evoked potentials (VEPs) (Nakanishi et al., 2017). However, although the current v-BCI has achieved superior performance (Han et al., 2023), it is always applied in a laboratory environment. The weak friendliness of human-computer interaction is an important reason, which limits BCIs’ daily use in real life (Miao et al., 2020). For most traditional v-BCIs, the stimulus blocks and instructions are spatially overlapping. Therefore, they commonly need users to stare at the irritating flash stimulus to output commands, which inevitably brings out visual fatigue for users (Chen et al., 2015). In addition, most traditional v-BCIs commonly used large-size or a relatively large numbers of visual stimuli to attract users’ attention and elicit distinct EEG features (Xu et al., 2018), which further brings a strong burden on users.

Recently, lateralized visual stimuli away from the central field of view has gained considerable interest in BCI studies because of the theory of retinotopic mapping (Yoshimura et al., 2011; Chen et al., 2017). According to retina-cortical mapping, the spatial pattern of VEPs is closely related to the position of visual stimuli in the visual field (Wurtz and Kandel, 2000). That is, there is a spatial mapping relation between the position of visual stimuli and the gaze position. Therefore, retina-cortical-based BCIs always arrange lateralized visual stimuli spatially separated from instructions, which no longer request subjects to stare at the irritating flash stimulus and therefore reduce the visual burden on the users. However, stimuli away from instructions may evoke EEG responses with lower SNR and discriminability. To address this problem, current retina-cortical-based BCIs commonly increase the size or number of visual stimuli, which brings out visual burden on users. For instance, in 2018, Chen accomplished a four-instructions speller that identified by a single motion stimulus (Chen et al., 2018). The instructions were fixed around a vertical bar (6°×0.32°), which appeared from left border of the central square and moved rightward. However, the size of the sliding stimuli had eccentricity with 6°, even larger than the 4°×4° of the stimulation block in traditional v-BCIs (Chen et al., 2015). In the same year, we developed a 32-command BCI system using very small lateral visual stimuli, and each stimuli only subtended 0.4° of the visual angle (Xu et al., 2018). The results of this study firstly demonstrated that the weak spatial evoked related potentials (ERPs) induced by small visual stimuli were also detectable for BCIs. For each command in this study, there was a set of left and right lateral visual stimuli flicking in different time sequences. That means two stimuli were used to code one command, which increased coding time and experimentation time. In 2021, we further designed a 16-command ERP-BCI with only one small visual stimulus. In this study, the spatial ERPs induced by a small visual stimulus was highly related to the distances and directions between the stimulus and the 16 gazed positions (Zhou et al., 2021). Inspired by these results, we believe that there is huge potential to design user-friendly and multi-instructions ERP-BCIs using tiny lateralized ERPs.

In this study, we designed a novel 3×3 ERP-BCI paradigm using small and lateralized visual stimuli, and the weak stimuli located between instruction rows/columns flashed in a row-column paradigm. Each of the stimuli had a small size with 0.4° of visual angle. Meanwhile, this paradigm used only three visual stimuli to code nine instructions, which reduced intensity of single-round stimulation. Furthermore, we conducted offline and online experiments with this novel paradigm, and used a template-matching method to recognize weak ERPs.

## 2. Method and materials

### 2.1. Participants

Nine healthy volunteers from Tianjin University (five males and four females, aged from 20 to 25 years) with normal or corrected-to-normal vision participated in the offline and online experiments. They had read and signed informed consents which were approved by the Research Ethics Committee of Tianjin University before the experiment.

### 2.2. Experimental procedure

Before the experiment, subjects were seated 70 centimeters in front of the screen and they were told to try their best to avoid behavior such as blinking and body shaking during the experiment. The paradigm was presented on a 24-inch LCD computer monitor with a 1920 × 1080-pixel resolution and a 120 Hz refresh rate. During the experiment, the subjects were told only need to stare at the instruction rather than flickering stimuli, and their eyes were always at the same height as the center of the paradigm.

**Figure 1:**
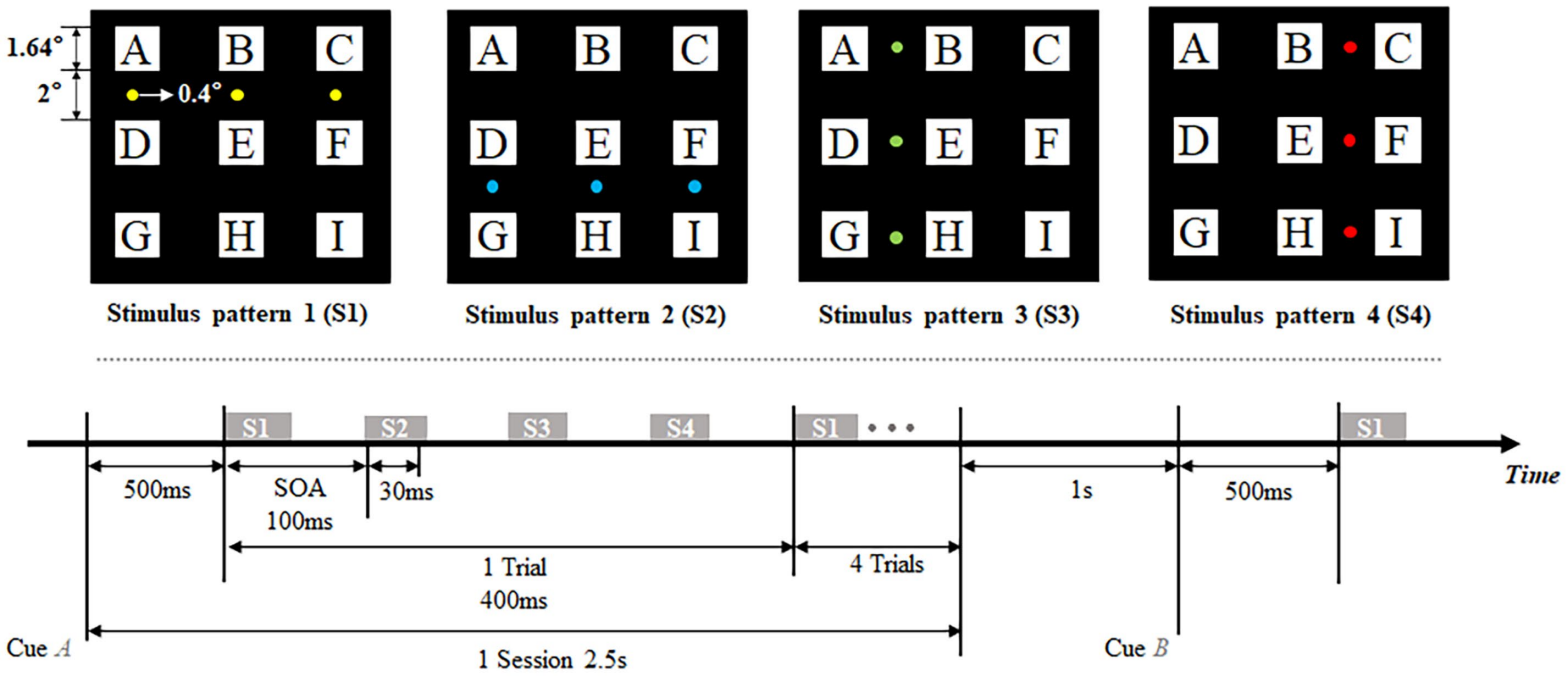
Stimulus patterns (top) and flowchart of experiment (bottom).

Four stimulus patterns of the offline experimental procedure are shown in the top panel of Figure 1. A 3 × 3 (each with a visual field of 1.64°× 1.64°) character matrix was presented in the center of the screen. Three small visual stimuli which only subtended 0.4° of visual angle were located between instruction rows/columns and flashed in the row-column paradigm in the order of four stimulus patterns. Each of four kinds of stimulus patterns were composed of three stimuli, and were tagged with S1, S2, S3, and S4, respectively. These four patterns divided the character matrix into four areas (upper, lower, left and right) in the paradigm. The flowchart of the offline experiment is shown in the bottom panel of Figure 1. In this experiment, one session was divided into the stage of cue and the stage of flicker. Cueing duration lasted 500 ms for subjects to shift their point of view, subjects were told to stare at the characters cued with a specific symbol ‘∇’. Flashing process consisted of 5 trials in offline experiment. Each trial consisted of 4 specific stimulus patterns which corresponded with the stimulus shown in the top panel of Figure 1. The stimulus onset asynchrony (SOA) between two consecutive stimulation was fixed at 100 ms, including 30 ms presentation of stimulation and 70 ms blank without stimulus. Thus, one trial lasted 400 ms that consisted of four stimuli, and the process of flashing lasted 2 s that consisted of 5 trials. In addition, the inter-session interval was fixed at 1 s, which allowed subjects’ activities such as blinking and avoided interference between each session.

In the offline experiment, data of 30 sessions were collected for each instruction which led to an experimental duration of 30 min for each subject. Subjects were told to take a short break to reduce visual fatigue between consecutive sessions. After the offline experiment, an online spelling task of randomly spelling nine characters twice was conducted to further evaluate the performance of the proposed ERP-BCI. All subjects were asked to make opinions on the comfort level of the proposed paradigm after completing character spelling.

### 2.3. Signal recording and pre-processing

In this study, the EEG data was recorded by the Neuroscan Synamps2 system with 64 electrodes. One referenced electrode was in the central area near Cz and one ground electrode was on the frontal lobe. The sampling rate of the system was set to 1,000 Hz and a 50 Hz notch filter was applied during the EEG acquisition. In the pre-processing, the EEG was firstly re-referenced to the average value of left and right mastoids M1, M2, and then filtered at 1–15 Hz by Chebyshev II filters. To make the data easy to calculate and store, the filtered EEG was down sampled at 500 Hz. This study used MetaBCI for realizing real-time high-speed data retrieving, data offline/online processing and output feedback. MetaBCI is a one-stop software for the construction of BCIs which was proposed as the first open-source software for BCIs in China.^1^

### 2.4. Classify algorithm of character-by-character recognition

It has been demonstrated that Discriminative Spatial Pattern (DSP) could effectively identify the spatial feature of EEG evoked by very small stimuli (Xu et al., 2018; Zhou et al., 2021). Thus, this study used DSP for extracting and recognizing EEG features, which consisted of two major parts: (1) the construction of DSPs and (2) pattern matching. It is well known that DSP finds a projection matrix W, which extracts the spatial features and removes the common noise from EEG (Duda et al., 2001). The projection matrix W can be obtained by maximizing the Fisher’s linear discriminant criterion as follows (Duda and Hart, 2006):


(1) $$J\left(W\right)=\frac{\left|S_{B}\right|}{\left|S_{W}\right|}=\frac{\left|W^{T}S_{B}W\right|}{\left|W^{T}S_{W}W\right|}\;$$

where $S_{B}$ indicates between-class scatter matrix and $S_{W}$ indicates within-class scatter matrix (Liao et al., 2007). In the proposed paradigm, distinguishable neural response patterns could be elicited when gazing at each of nine instructions in each stimulus pattern. In other words, nine instructions could be recognized in each of four stimulus patterns. Therefore, we calculated projection matrix $W_{K}^{i}\in R^{N_{C}\times N_{C}}\;\left(i=1\ldots 4,K=1\ldots 9\right)$ of each stimulus patterns and then obtained training template ${\hat{X}}_{K}^{i}\in R^{N_{C}\times N_{T}}\;$after filtering of noise removal, where $W_{K}^{i}$ indicates the projection matrix of instruction K from stimulus pattern i and ${\hat{X}}_{K}^{i}$ is the average of training samples from the stimulus pattern i and instruction K, $N_{C}$ is the number of channels and $N_{T}$ is the number of time points.

In pattern matching, test sample $Y\in R^{N_{C}\times N_{T}}$ can be divided into four segments $Y_{i}$ according to the time windows of four stimulus patterns. Each segment data can be template-matched according to its stimulus pattern, then the decision value of specific characters ${\tilde{\rho }}_{K}$ can be calculated by the $W_{K}^{i}{}^{T}{\hat{X}}_{K}^{i}$ and $Y^{i}W_{K}^{i}$ as follows:


(2) $$\begin{array}{c} {\tilde{\rho }}_{K}=\overset{4}{\underset{i=1}{\sum }}\rho_{i}=\overset{4}{\underset{i=1}{\sum }}corr\left(W_{K}^{i}{}^{T}{\hat{X}}_{K}^{i},Y^{i}W_{K}^{i}\right)\; \end{array}$$

And the predicted instruction of Y is:


(3) $$\begin{array}{c} \tilde{k}=\max\;\left\{\;{\tilde{\rho }}_{K},K=1,2,..,9\;\right\}\; \end{array}$$

### 2.5. Information transfer rate

To further evaluate the performance of the proposed system, this study investigated the Information Transfer Rate (Wolpaw et al., 2000), which is defined as


(4) $$\begin{array}{c} ITR=\left(\log_{2}N+P\log_{2}P+\left(1-P\right)\log_{2}\frac{1-P}{N-1}\right)\times \left(\frac{60}{T}\right)\; \end{array}$$

where N is the total number of system commands, P is the accuracy of target identification and *T* is the consuming time for each command outputting, which includes 500 ms for the subjects to shift their focus additionally.

## 3. Results and analysis

### 3.1. Analysis of ERPs with spatial specificity in the novel paradigm

**Figure 2:**
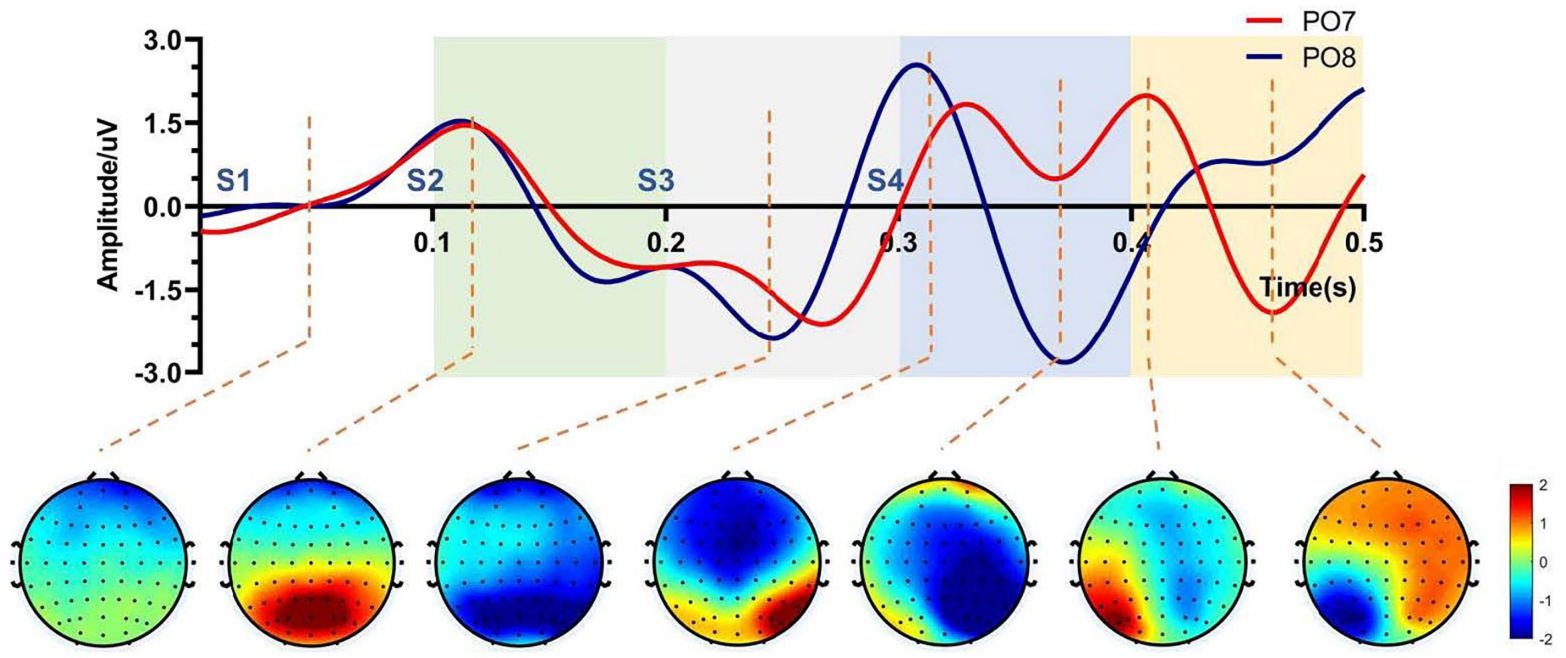
Spatial response of character E in one trial, which contains four stimulus patterns S1, S2, S3, and S4. Four colors represent timing windows of spatial responses for four stimulus patterns.

Previous studies indicated that spatial features of ERPs commonly appeared at 100–200 ms after the presentation of stimuli (Chen et al., 2018; Xu et al., 2018; Zhou et al., 2021). For one trial arranged in this paradigm, various stimuli presented at 0 ms, 100 ms, 200 ms, and 300 ms corresponding to four stimulus patterns S1, S2, S3, and S4 individually (Figure 1). Thus, timing windows of ERP responses, respectively, concentrated in 100–200 ms, 200–300 ms, 300–400 ms, and 400–500 ms corresponding to each of four stimulus patterns in one trial. In Figure 2, code ‘E’ among all characters was firstly chosen for analysis as its position which has an equal visual angle from all stimulus patterns. We first investigated waveforms and typical topographies for code ‘E’ during 0–500 ms after the presentation of visual stimuli across nine subjects. ERP features at electrodes of PO7 and PO8 were analyzed because of their stronger and more typical spatial responses than other electrodes (Chen et al., 2018). As shown in Figure 2, ERP responses reached the first wave crest around 120 ms that evoked by S1. Meanwhile, most signals in the central occipital region presented a positive amplitude in the topography at that moment, which is generally acknowledged as the responses evoked by stimulus located above the line of sight. Subsequently, during 200–300 ms, potentials showed a negative variation, and obvious negative waves could be observed near 230 ms. Most signals in the central occipital region presented a negative amplitude in the topography at that moment. The polarities of most electrodes were observed inverted from 100–200 ms to 200–300 ms, which corresponded to ERP responses for S1 and S2, respectively. Then during 300–400 ms, ERP responses were concentrated in the right hemisphere of the brain in the view of the topography and the waveform at PO8. A laterally symmetric response could be found during 400–500 ms after S4, which were concentrated in the left hemisphere of the brain. The ERP responses with spatial specificity appeared after S3 and S4 showed contralateral maximal responses when the stimuli were located on the left of the visual field (stimulus pattern 3) and right of the visual field (stimulus pattern 4).

**Figure 3:**
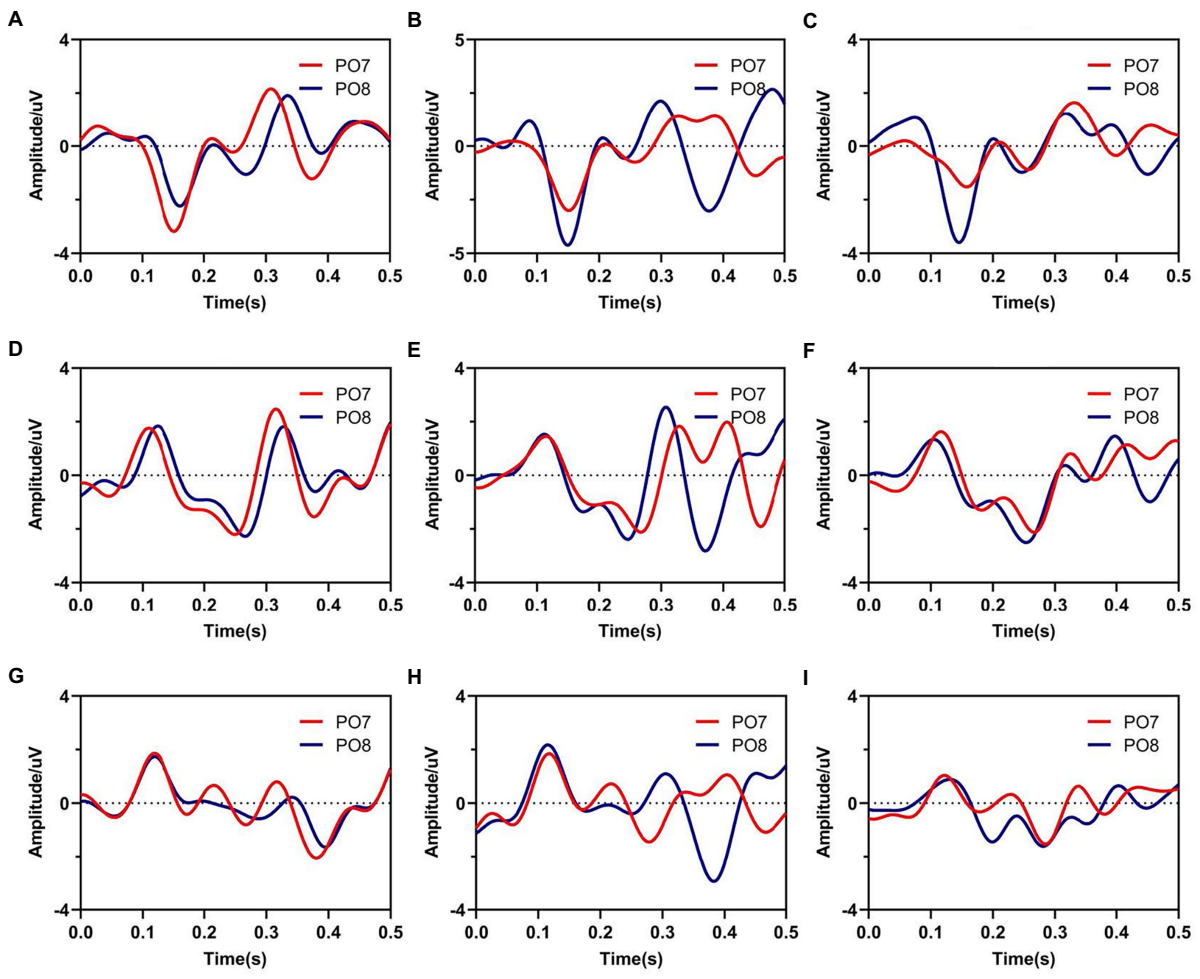
Average waveforms from electrodes of PO7 and PO8 when subjects gazed at nine instructions. Subfigures **(A–I)** correspond to real characters **(A–I)** respectively.

Furthermore, Figure 3 showed average waveforms across all subjects when gazing at each of nine instructions. For a clear presentation of results, the location of subgraphs in Figure 3 referenced the real position of the instruction in the character matrix in paradigm. It could be observed that the spatial features of ERPs at PO7 and PO8 were similar when the subjects noted the specific characters in the same time window of S1 and S2. For characters A, B and C in the top row, stimuli were presented below the instruction in pattern of S1, and spatial responses concentrated during 100–200 ms after the presentation of visual stimuli, meanwhile, electrodes of PO7 and PO8 showed negative potentials and peaked around 170 ms. For characters D, E, F, G, H, and I in the middle and bottom row, stimuli were presented above the instruction in pattern of S1, the polarities of PO7 and PO8 were observed inverted in the same time window. These results were highly related to the position between instructions and visual stimuli. For stimulus pattern 1, visual stimuli divided the character matrix into upper and lower parts. Thus, visual stimuli were commonly located in the upper or lower visual field, which resulted in polarity-inverted when subjects gazed at some characters. Similarly, visual stimuli of stimulus pattern 2 also divided the character matrix into upper and lower parts. For responses of S2 during 200–300 ms, characters A, B, C, D, E and F in the top and middle row had similar waveform variation, which was different from characters G, H and I. For stimulus patterns 3 and 4 (EEG responses during 300–400 ms and 400–500 ms), visual stimuli divided the character matrix into left and right parts. Thus, visual stimuli were commonly located in the left or right visual field, which resulted in the lateralization spatial responses of the brain.

**Figure 4:**
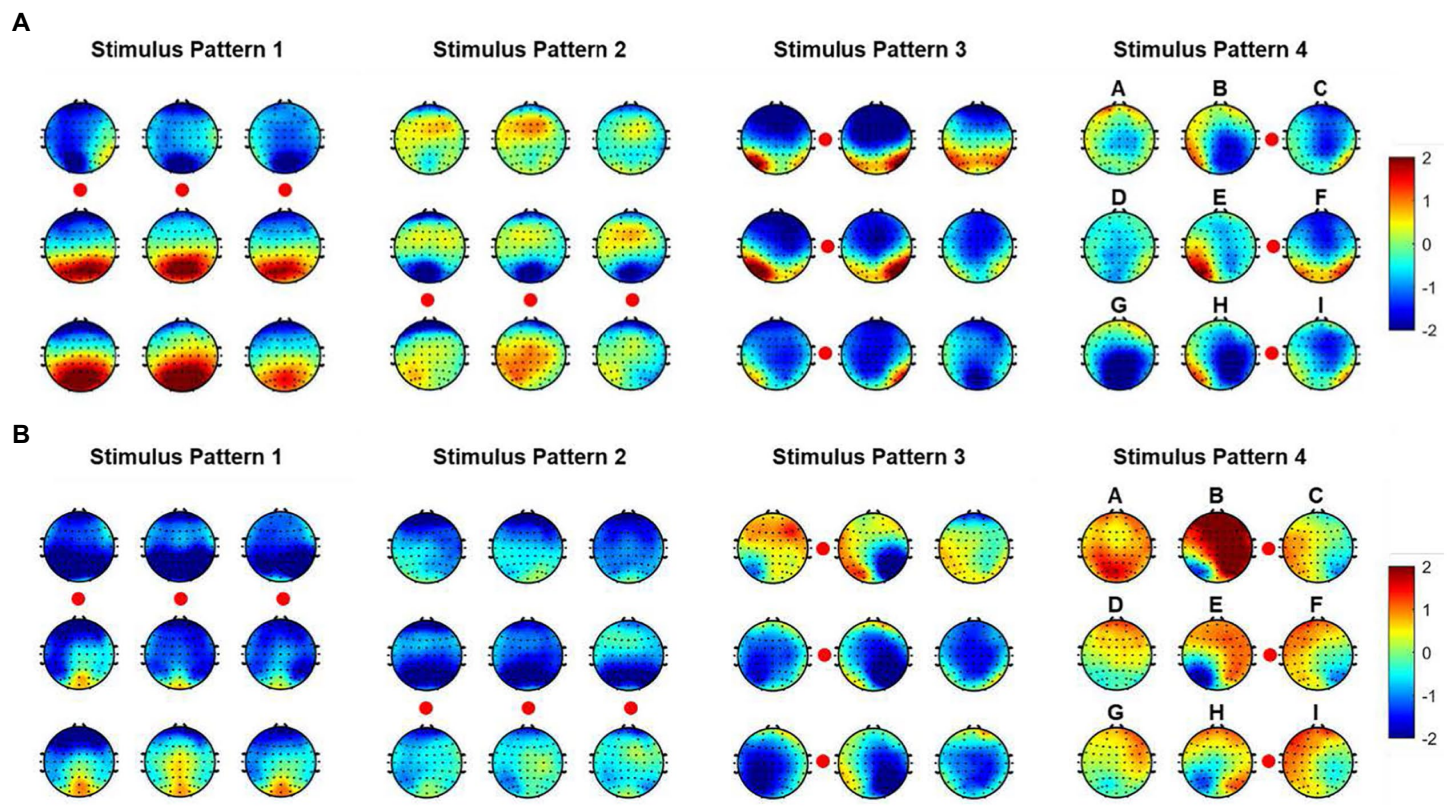
Spatial responses at 120 ms and 170 ms after stimulation of each stimulus patterns. Nine topographies correspond to nine instructions, and the red circles correspond to the stimulation locations. **(A)** Spatial responses at 120 ms. **(B)** Spatial responses at 170 ms.

In order to indicate the characteristic of spatial responses for all instructions under four stimulus patterns, we further explored the brain topographies elicited by four stimulus patterns. Figure 4 showed the spatial responses at 120 ms and 170 ms after the stimulation of each stimulus pattern, because Figure 3 showed that the responses of those moments had stronger responses after one stimulation. For stimulus patterns 1 and 2, visual stimuli divided the character matrix into upper and lower parts, and the amplitude of most electrodes in the occipital region was reversed in polarity when subjects gazed at corresponding parts. The phenomenon was more significant at 120 ms but weak at 170 ms after the presentation of stimulation. For stimulus patterns 3 and 4, the visual stimuli divided instructions into left and right parts. When visual stimuli were presented to the left or right sides of instructions, maximum responses were elicited in the contralateral hemisphere, which could be found at both 120 ms and 170 ms. At 120 ms, most electrodes of the occipital region performed lateralization of positive potentials, while at 170 ms, they performed negative potentials.

In addition, characters with similar positions around stimuli showed similar spatial responses. This was consistent with the performance of average waveforms shown in Figure 3. For example, in stimulus pattern 1 and stimulus pattern 2, which divided instructions into upper and lower parts, instructions D, E, and F performed similar spatial responses, as were located on the upper (lower) side of the visual stimulus at the same time. While in stimulus pattern 3 and stimulus pattern 4, which divided instructions into left and right parts, characters B, E, and H showed similar spatial responses. In addition, it could be observed that the strength of spatial responses decreased with increasing distance between instructions and visual stimuli as shown in Figure 4.

Our analysis indicated that when gazing at different characters, ERPs with different spatial specificity could be evoked by four stimulus patterns which were composed of three tiny stimuli. Meanwhile, the characteristic of spatial response was mainly concentrated in the parietal and occipital region, which provided basic reference to electrode selection.

### 3.2. Classification results for different stimulus patterns

**Figure 5:**
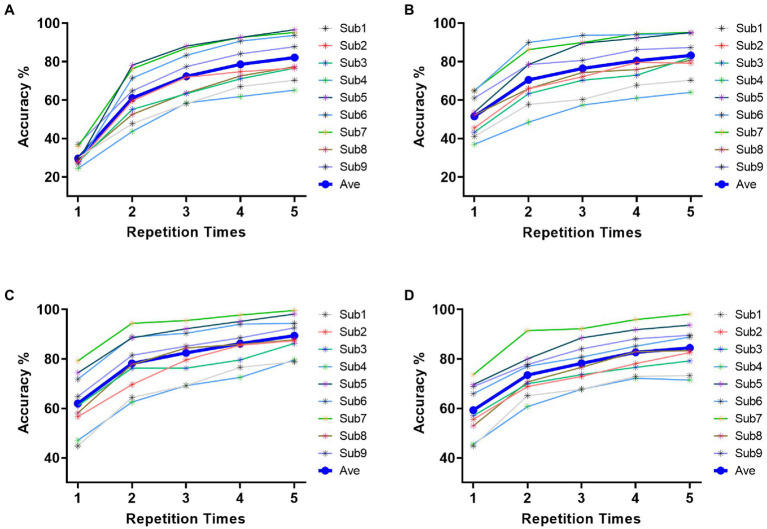
Classification accuracy across all subjects of four stimulus patterns. **(A)** classification accuracy of stimulus pattern 1. **(B)** classification accuracy of stimulus pattern 2. **(C)** classification accuracy of stimulus pattern 3. **(D)** classification accuracy of stimulus pattern 4.

Based on the results of feature analysis in section 3.1, 21 electrodes (P1, P3, P5, P7, Pz, P2, P4, P6, P8, PO3, PO5, PO7, POz, PO4, PO6, PO8, O1, Oz, O2, CB1, CB2) in parietal and occipital region were selected for target identification. Figure 5 indicated the classification results for each of four stimulus patterns based on DSPs. The horizontal coordinate represents the repetition times of trials utilized in pattern matching. The nine-character classification accuracy increased with the repetition times for all stimulus patterns. Specifically, as shown in Figure 5A, the average accuracy of stimulus pattern 1 was 29.63%, 61.07%, 72.39%, 78.60%, 82.18% for 1 to 5 repetitions, respectively. The highest individual accuracy achieved 96.67% for Sub5 at 5 repetitions. In Figure 5B, the average accuracy of stimulus pattern 2, respectively, achieved 51.56%, 70.53%, 76.54%, 80.49%, 83.25% for 1 to 5 repetitions with the highest individual accuracy of 95.19% for Sub7 at 5 repetitions. For Figures 5C,D, the average accuracy performed higher than the accuracy in Figures 5A,B, which achieved 89.42 and 84.49% after 5 repetitions, respectively. Overall, grand-accuracy of all stimulus patterns for 1 to 5 repetitions were higher than 11.11% (random level), which further demonstrated the divisibility of 9 characters in each of stimulus patterns.

### 3.3. Classification results for target identification in the novel paradigm

**Figure 6:**
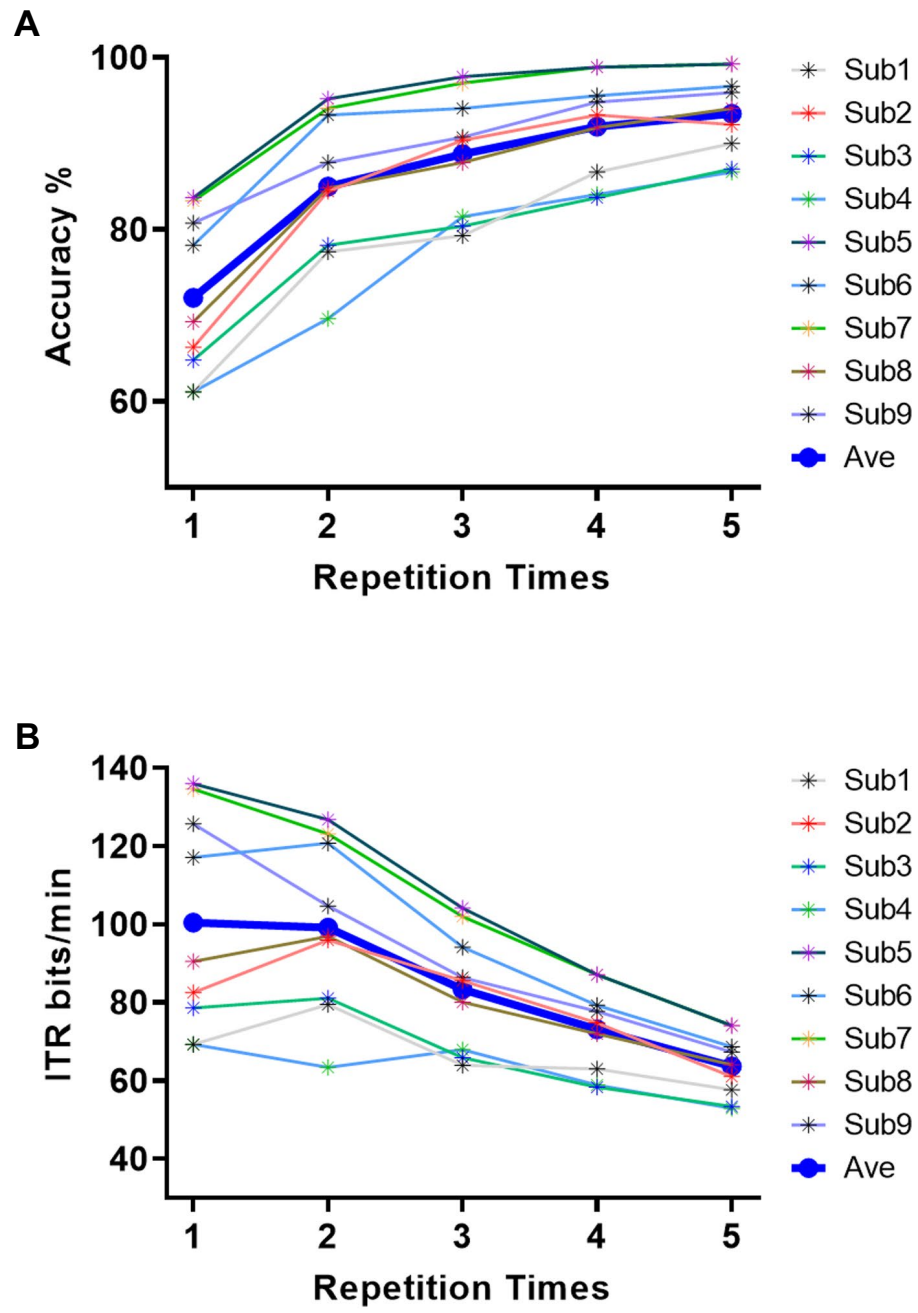
Performance for all subjects in offline experiment. **(A)** Character accuracy across all subjects. **(B)** ITR across all subjects.

In pattern matching for target identification of nine-character matrix, classification results of four stimulus patterns in section 3.2 were employed to joint determine the specific instruction. During the process of target character identification, we constructed templates and spatial filters from training set for each of nine characters under each condition of four stimulus patterns, then used pattern matching to calculate the correlation between the template and test sample after spatial filtering, and lastly confirm the target character based on formula (2) and (3). Figure 6A showed the classification accuracies of all subjects in the offline experiment. The horizontal coordinate represents the repetition times of trials employed in classification, which corresponds to stimulus durations of 0.4 s, 0.8 s, 1.2 s, 1.6 s, and 2 s, respectively. It could be observed that the character accuracies increased with the repetition times, the average classification accuracies across nine participants were 72.06%, 84.98%, 88.77%, 91.97%, and 93.46% for 1 (0.4 s stimulation duration) to 5 (2 s stimulation duration) repetition times. Most subjects performed well with accuracies between 60% and 100%, subject 5 reached the highest classification accuracy among all subjects of 83.7%, 95.19%, 97.78%, 98.89%, and 99.26% for 1 to 5 repetition times. Seven of nine subjects could achieve accuracy over 90% and four subjects achieved accuracy over 95%. Figure 6B showed offline character ITR for all subjects. The consuming time for each command outputting was composed of cue (0.5 s) and stimulus duration corresponding to repetition times. The average ITRs across nine subjects were 100.39 bits/min, 99.12 bits/min, 83.31 bits/min, 73.11 bits/min, and 63.65 bits/min for 1 to 5 repetition times. Among all subjects, the maximal ITR achieved 135.97 bit/min for Sub5 for 1 repetition time. Notably, ITRs of four subjects out of nine achieved over 120 bits/min, which suggested the proposed v-BCI has the potential for high online performance.

To further evaluate the feasible of the proposed v-BCI, an online task of randomly spelling all characters was set up. For each subject, online repetition times were firstly determined according to the highest ITRs in the offline experiments. Then online test of randomly spelling all instructions twice was notified to each subject. Table 1 showed the performance of online test. It can be illustrated that all subjects performed 1–2 repeated trials for one character selection. The average online ITR could achieve 120.95 bits/min, meanwhile, the highest ITR was 177.5 bits/min (Sub5) and the lowest ITR was 69.28 bits/min (Sub4) in the experiment. The online results were similar to that of the offline experiment. ITRs of five of nine subjects achieved over 100 bits/min in the online test and all of the subjects achieved over 60 bits/min, which demonstrated the feasible of proposed v-BCI. These results demonstrated that this novel paradigm using small and few stimuli has the potential to achieve high performance BCI.

**Table 1:** Performance of online spelling.

	Repetition times	Accuracy %	ITR bits/min
Sub1	2 (0.5 + 0.8 s)	83.33	92.41
Sub2	2 (0.5 + 0.8 s)	83.33	92.41
Sub3	2 (0.5 + 0.8 s)	94.44	122.88
Sub4	1 (0.5 + 0.4 s)	61.11	69.28
Sub5	1 (0.5 + 0.4 s)	94.44	177.5
Sub6	2 (0.5 + 0.8 s)	100	146.3
Sub7	1 (0.5 + 0.4 s)	88.89	156
Sub8	2 (0.5 + 0.8 s)	83.33	133.48
Sub9	1 (0.5 + 0.4 s)	72.22	98.29
Ave	–	84.56	120.95
Std	–	12.04	35.43

### 3.4. Fatigue level of subjects

Notably, we asked subjects for their opinions on the comfort level of the proposed paradigm. All of them considered the v-BCI performed friendly both in interaction comfort and performance. Table 2 showed the fatigue level (score: 1–5) by all subjects with an average score of 1.89. The highest fatigue score was 4 (Sub5) while the lowest score was 1 (Sub3, Sub4, and Sub6). These results demonstrated that the proposed paradigm has a friendly interaction mode. Compared with traditional v-BCI, the stimulus presented in our study was more natural for subjects to use, which made it possible to use out of the laboratory environment.

**Table 2:** Fatigue level of the proposed paradigm.

Subjects	Fatigue level score
Sub1	2
Sub2	2
Sub3	1
Sub4	1
Sub5	4
Sub6	1
Sub7	2
Sub8	2
Sub9	2
Ave	1.89
Std	0.93

## 4. Discussion

Traditional v-BCIs such as P300-spellers (Farwell and Donchin, 1988; Jin et al., 2011) and SSVEP-BCIs (Speller, 2015; Jiao et al., 2018) have achieved high ITR performance in previous studies. However, the unfriendly interactive mode of strong and complicated stimuli hinders their practical usage in real life. To address this problem, our study proposed a novel v-BCI which only used three weak stimuli to accomplish nine instructions controlling. The average classification accuracy and ITR in the online spelling test achieved 84.56% and 120.95 bits/min across nine subjects, which suggested the proposed paradigm was a candidate for friendly v-BCI. From the perspective of practical application of the v-BCI, this study still exists something for improving the performance of the system.

**Figure 7:**
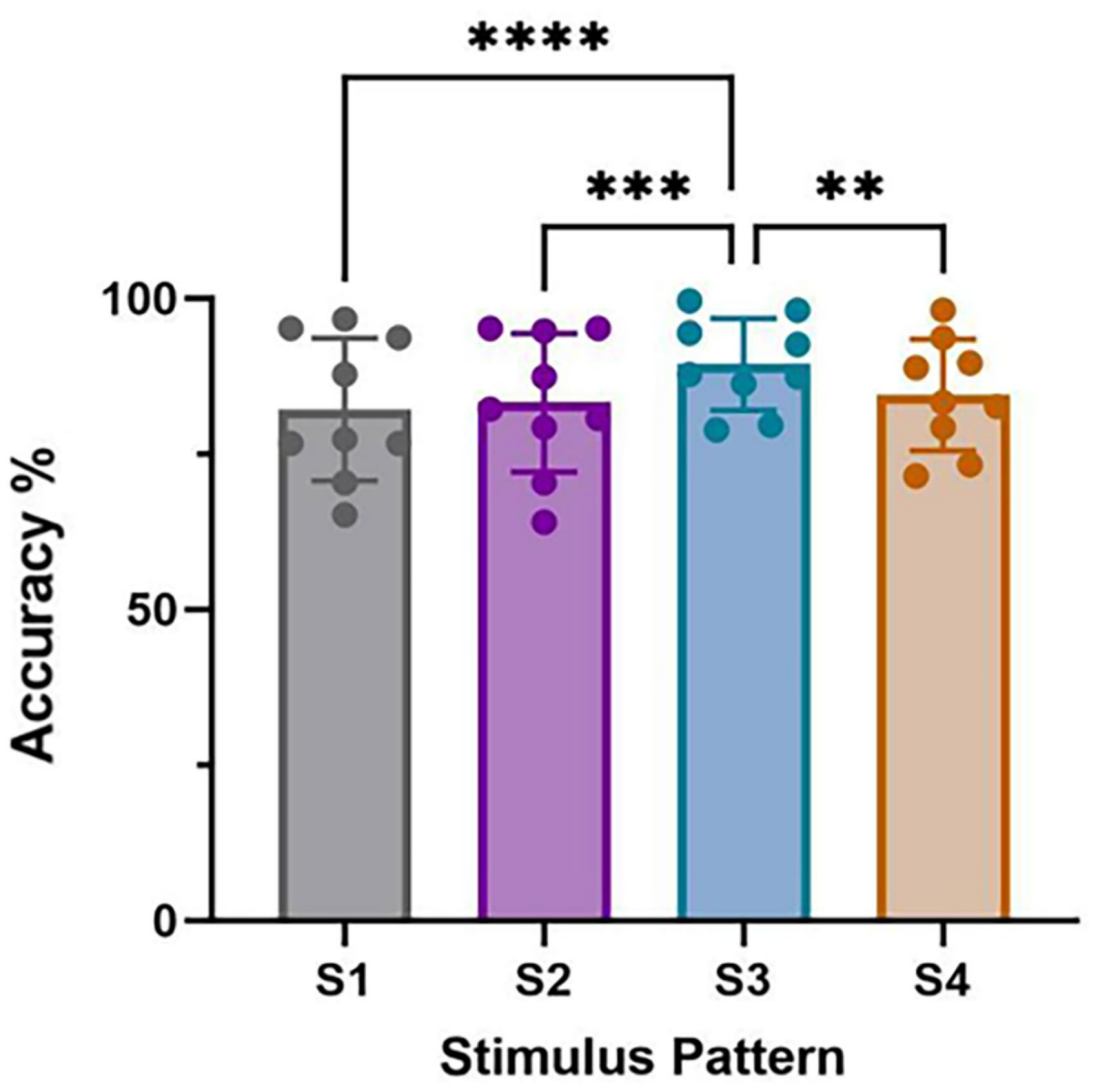
Classification accuracy of nine characters for four stimulus patterns at 5 repetitions. (****p < 0.0001, ***p < 0.001, **p < 0.01).

On the one hand, paradigm designing was novel and effective, which could be further optimized in future studies. We have demonstrated the feasibility of character identification in each stimulus pattern and then employed these results for joint decisions. We investigated the performance for four stimulus patterns at 5 repetitions in Figure 5 and performed one-way repeated measures ANOVAs on classification accuracies among four stimulus patterns. As shown in Figure 7, the results manifested that there was a significant difference in character accuracy among different stimulus patterns (*F* (3, 24) =13.52, *p* < 0.0001). The accuracy of S3 was significantly higher than S1 (*p* < 0.0001), S2 (*p* < 0.001), and S4 (*p* < 0.01), meanwhile, among S1, S2, and S4, there was no significance of classification accuracy. According to these results, we could consider the superiority of stimulus pattern 3 in paradigm designing. Moreover, although there was no significance between S4 and the other patterns, the accuracy of it was still higher than S1 and S2. This may be attributed to the difference between left and right hemispheres of the brain is higher than that between upper and lower hemispheres (Zhou et al., 2021).

**Figure 8:**
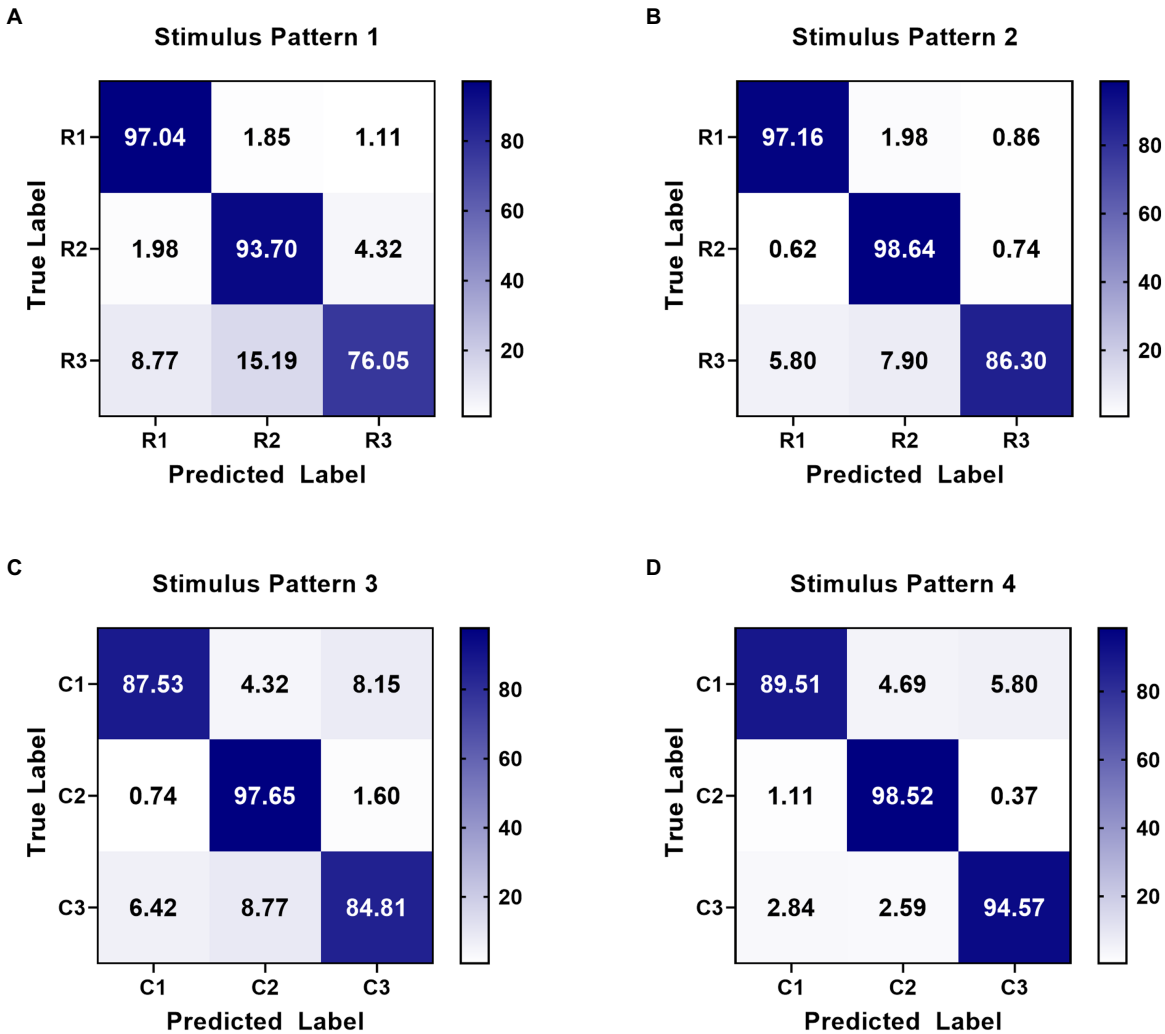
Confusion matrices of row-column classification at 5 repetitions for four stimulus patterns. **(A)** Confusion matrix for row identification (R1, R2, and R3) in stimulus pattern 1. **(B)** Confusion matrix for row identification (R1, R2, and R3) in stimulus pattern 2. **(C)** Confusion matrix for column identification (C1, C2, and C3) in stimulus pattern 3. **(D)** Confusion matrix for column identification (C1, C2, and C3) in stimulus pattern 4.

**Figure 9:**
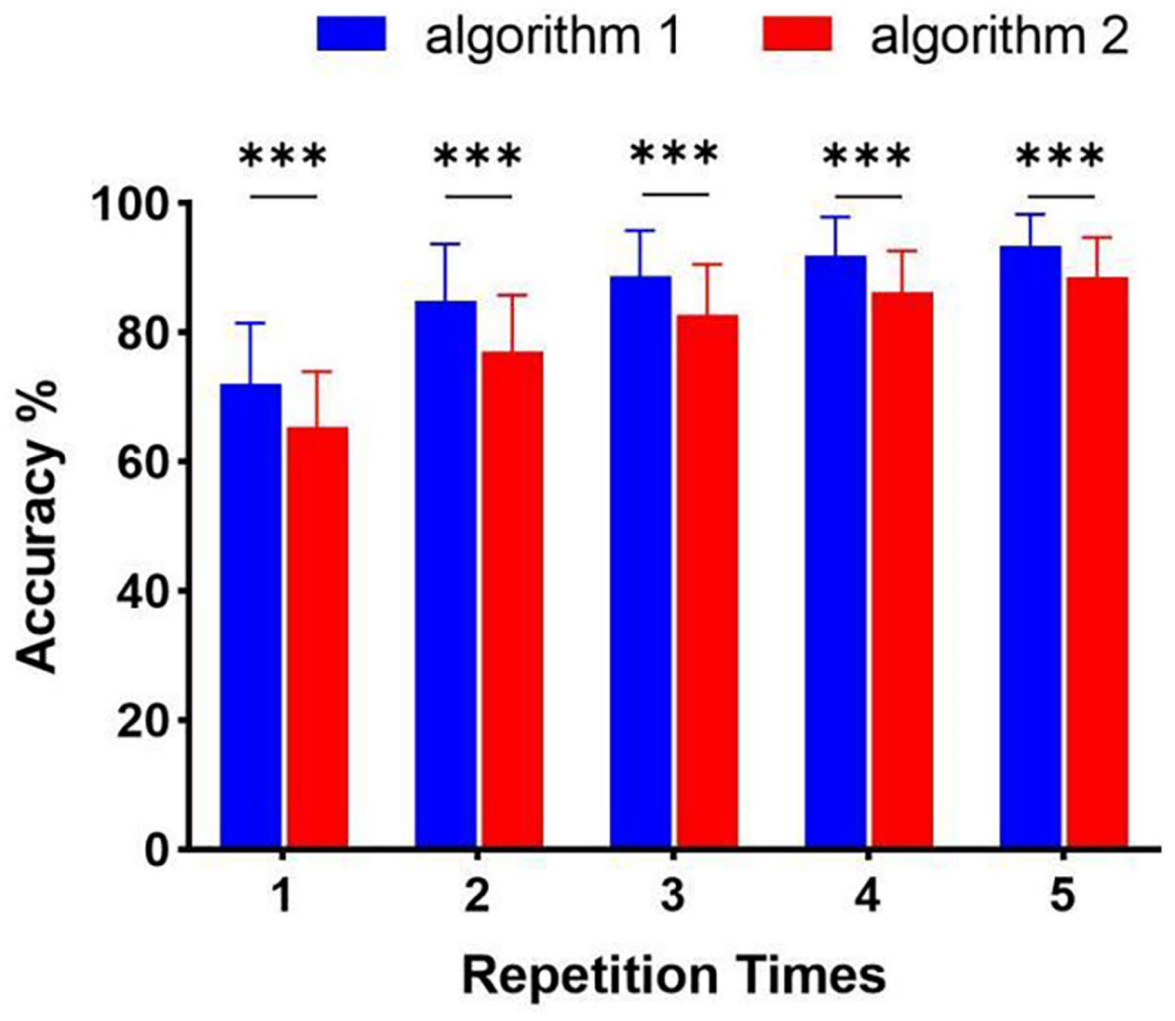
Offline classification accuracy of algorithm 1 (recognition of character-by-character) versus algorithm 2 (recognition of row-column) (***p < 0.001).

On the other hand, the classification method aimed at this paradigm achieved high performance in online spelling. However, this method still has limitations when applied in complex environments. Specifically, when the number of system instructions becomes larger, the decoding strategy for each character in every situation has low efficiency because of constructing more templates and spatial filters. Thus, decreasing the number of decoding templates becomes significant. For this paradigm using characteristic of spatial features, the relative position and distance between visual stimuli and the gazed character are two vital aspects of paradigm designing (Zhou et al., 2021). According to the analysis of spatial features in Figure 4, distinct spatial features of EEG were evoked by four stimulus patterns. The spatial responses of the gazed character located in rows or columns under four stimulus patterns were similar. Thus, we could use some instructions to joint construct one template according to their relative position and distance between stimuli and instruction under different stimulus patterns. For stimulus pattern 1 and 2, the visual stimuli divided instructions into three rows (A, B, C), (D, E, F), and (G, H, I), which performed similar spatial responses in the analysis of spatial features. We tag these rows as R1, R2, and R3. For stimulus pattern 3 and pattern 4, the visual stimuli divided instructions into three columns (A, D, G), (B, E, H) and (C, F, I), which were tagged as C1, C2, and C3. We first investigated the divisibility of row or column sequences under four stimulus patterns. As shown in Figure 8, the classification results showed variability between rows or between columns. For identification of rows in S1 and S2, R3 performed easily mixable with R1 and R2. This was possibly due to the position of the gazed character located underneath the stimuli, which was consistent with previous studies (Chen et al., 2018; Zhou et al., 2021). For identification of columns in S3 and S4, the average accuracies were lower than rows, however, all identification accuracies of rows and columns were much higher than the random level for three-target classification (33%), which demonstrated the feasibility of employing row/column identification for one target recognition. Based on these results, each instruction of this 3 × 3 character matrix could be determined by decoding rows or columns after four times of stimulus using four stimulus patterns, which was similar to the classical classification strategy used in P300-speller. The average accuracies across nine participants were 65.43%, 77.08%, 82.67%, 86.26%, and 88.64% for 1 to 5 repetitions using this new algorithm. The highest accuracy was 97.78% for Sub5 and Sub7 using 1 repetition of stimulus trial. To further evaluate the performance of this algorithm, Figure 9 shows the offline classification of two algorithms for recognition of each character (algorithm 1) vs. recognition of row-column (algorithm 1). One-way repeated measures ANOVAs were performed on classification accuracies between two algorithms. The results revealed that algorithm 1 performed better than algorithm 2 in each repetition time (*p* < 0.001). However, although the classification of algorithm 2 was lower, it can also achieve 88.64% at 5 repetitions, which showed great performance as well. In addition, algorithm 2 could significantly decrease the number of templates from 9 to 3, which showed great potential in a complex system with large instructions.

In addition to the advantages for above two aspects, this “weak” and “small amount” stimulus-based v-BCI also has a wider application prospect due to its friendliness of interaction, such as application of games based on BCIs. Meanwhile, the proposed BCI system could achieve high ITR performance in order to ensure that the game interaction is smooth.

## 5. Conclusion

This study proposed a novel v-BCI paradigm using weak and small number of stimuli to accomplish nine instructions controlling. The weak and few stimuli would reduce visual burden and experimental training time, meanwhile, it could also evoke ERPs with characteristics of both spatial and temporal features. A template-matching method based on DSPs was employed to recognize ERPs containing the intention of users. Results of offline and online experiments across nine subjects showed that the average accuracy of offline experiment was 93.46% and the highest online ITR achieved 177.5 bits/min, which demonstrated the feasibility to implement such a friendly v-BCI using this novel paradigm. Furthermore, the proposed paradigm achieved higher ITR than traditional ones using ERPs as the controlled signal, which showed its great potential of being widely used in various fields.

## Data availability statement

The original contributions presented in the study are included in the article/supplementary material, further inquiries can be directed to the corresponding author.

## Ethics statement

The studies involving human participants were reviewed and approved by Research Ethics Committee of Tianjin University. The patients/participants provided their written informed consent to participate in this study.

## Author contributions

XX and RG designed the study and wrote the manuscript. XZ collected the relevant literatures. MX and DM reviewed and edited the manuscript. All authors read and approved the submitted manuscript.

## Funding

This work was supported by the STI 2030—Major Projects 2022ZD0210200, National Natural Science Foundation of China (Nos. 62106170, 62122059, 81925020, and 62006014), and Introduce Innovative Teams of 2021 “New High School 20 Items” Project (2021GXRC071).

## Conflict of interest

The authors declare that the research was conducted in the absence of any commercial or financial relationships that could be construed as a potential conflict of interest.

## Publisher’s note

All claims expressed in this article are solely those of the authors and do not necessarily represent those of their affiliated organizations, or those of the publisher, the editors and the reviewers. Any product that may be evaluated in this article, or claim that may be made by its manufacturer, is not guaranteed or endorsed by the publisher.

## Footnotes

^1^ https://github.com/TBC-TJU/MetaBCI

## References

[ref1] Chen J. Li Z. Hong B. Maye A. Engel A. K. Zhang D. (2018). A single-stimulus, multitarget BCI based on retinotopic mapping of motion-onset VEPs. IEEE Trans. Biomed. Eng. 66, 464–470. doi: 10.1109/TBME.2018.2849102, PMID: 29993456

[ref2] Chen X. Wang Y. Nakanishi M. Gao X. Jung T.-P. Gao S. (2015). High-speed spelling with a noninvasive brain–computer interface. Proc. Natl. Acad. Sci. 112, E6058–E6067. doi: 10.1073/pnas.1508080112 26483479 PMC4640776

[ref3] Chen J. Zhang D. Engel A. K. Gong Q. Maye A. (2017). Application of a single-flicker online SSVEP BCI for spatial navigation. PLoS One 12:e0178385. doi: 10.1371/journal.pone.0178385, PMID: 28562624 PMC5451069

[ref4] Duda R. O. Hart P. E. (2006). Pattern classification. Hoboken, NJ: John Wiley & Sons.

[ref5] Duda R. O. Hart P. E. Stork D. G. (2001). Pattern classification. 2nd Edn, Hoboken, NJ: John Wiley & Sons. 58, 16.

[ref6] Farwell L. A. Donchin E. (1988). Talking off the top of your head: toward a mental prosthesis utilizing event-related brain potentials. Electroencephalogr. Clin. Neurophysiol. 70, 510–523. doi: 10.1016/0013-4694(88)90149-6, PMID: 2461285

[ref7] Han J. Xu M. Xiao X. Yi W. Jung T.-P. Ming D. (2023). A high-speed hybrid brain-computer interface with more than 200 targets. J. Neural Eng. 20:016025. doi: 10.1088/1741-2552/acb105, PMID: 36608342

[ref8] Jiao Y. Zhang Y. Wang Y. Wang B. Jin J. Wang X. (2018). A novel multilayer correlation maximization model for improving CCA-based frequency recognition in SSVEP brain–computer interface. Int. J. Neural Syst. 28:1750039. doi: 10.1142/S0129065717500393, PMID: 28982285

[ref9] Jin J. Allison B. Z. Sellers E. W. Brunner C. Horki P. Wang X.. (2011). An adaptive P300-based control system. J. Neural Eng. 8:036006. doi: 10.1088/1741-2560/8/3/036006, PMID: 21474877 PMC4429775

[ref10] Lebedev M. A. Nicolelis M. A. (2006). Brain–machine interfaces: past, present and future. Trends Neurosci. 29, 536–546. doi: 10.1016/j.tins.2006.07.004, PMID: 16859758

[ref11] Liao X. Yao D. Wu D. Li C. (2007). Combining spatial filters for the classification of single-trial EEG in a finger movement task. IEEE Trans. Biomed. Eng. 54, 821–831. doi: 10.1109/TBME.2006.889206 17518278

[ref12] Miao Y. Yin E. Allison B. Z. Zhang Y. Chen Y. Dong Y.. (2020). An ERP-based BCI with peripheral stimuli: validation with ALS patients. Cogn. Neurodyn. 14, 21–33. doi: 10.1007/s11571-019-09541-0, PMID: 32015765 PMC6974196

[ref13] Nakanishi M. Wang Y. Chen X. Wang Y.-T. Gao X. Jung T.-P. (2017). Enhancing detection of SSVEPs for a high-speed brain speller using task-related component analysis. IEEE Trans. Biomed. Eng. 65, 104–112. doi: 10.1109/TBME.2017.2694818, PMID: 28436836 PMC5783827

[ref14] Speller I. (2015). A dynamically optimized SSVEP brain-computer. I.E.E.E. Trans. Biomed. Eng. 62, 1447–1456. doi: 10.1109/TBME.2014.2320948 24801483

[ref15] Wolpaw J. R. (2014). The BCI endeavor and the mission of this new journal. Brain Comput. Interfaces 1, 2–4. doi: 10.1080/2326263X.2014.884740

[ref16] Wolpaw J. R. Birbaumer N. Heetderks W. J. McFarland D. J. Peckham P. H. Schalk G.. (2000). Brain-computer interface technology: a review of the first international meeting. IEEE Trans. Rehabil. Eng. 8, 164–173. doi: 10.1109/TRE.2000.847807, PMID: 10896178

[ref17] Wurtz R. H. Kandel E. R. (2000). Central visual pathways. Principles Neural Sci. 4, 523–545.

[ref18] Xu M. Qi H. Wan B. Yin T. Liu Z. Ming D. (2013). A hybrid BCI speller paradigm combining P300 potential and the SSVEP blocking feature. J. Neural Eng. 10:026001. doi: 10.1088/1741-2560/10/2/026001, PMID: 23369924

[ref19] Xu M. Xiao X. Wang Y. Qi H. Jung T.-P. Ming D. (2018). A brain–computer interface based on miniature-event-related potentials induced by very small lateral visual stimuli. IEEE Trans. Biomed. Eng. 65, 1166–1175. doi: 10.1109/TBME.2018.2799661, PMID: 29683431

[ref20] Yoshimura N. Itakura N. Fazel R. (2011). Usability of transient VEPs in BCIs. Recent Adv. Brain Comput. Interface Syst., 119–134. doi: 10.5772/14171

[ref21] Zhou X. Xu M. Xiao X. Wang Y. Jung T.-P. Ming D. (2021). Detection of fixation points using a small visual landmark for brain–computer interfaces. J. Neural Eng. 18:046098. doi: 10.1088/1741-2552/ac0b51, PMID: 34130268

